# Is Cross‐Matching Required? Analysis of Blood Transfusion in Gynecological Surgeries at an Academic Hospital in Bangkok, Thailand: A Cross‐Sectional Study

**DOI:** 10.1002/hsr2.72086

**Published:** 2026-03-30

**Authors:** Chutima Topipat, Punyanuch Chulnoul

**Affiliations:** ^1^ Department of Obstetrics and Gynecology, Faculty of Medicine Ramathibodi Hospital Mahidol University Bangkok Thailand; ^2^ Obstetrics and Gynecology Operating Room, Nursing Service Department, Faculty of Medicine Ramathibodi Hospital Mahidol University Bangkok Thailand

**Keywords:** blood transfusion, blood utilization, cross‐matching, gynecological surgeries

## Abstract

**Background and Aims:**

Effective utilization of perioperative blood transfusions can reduce costs and minimize blood waste. This study aimed to audit the appropriateness of current blood utilization practices in gynecological surgeries, characterize transfusion rates, and identify associated risk factors for transfusion.

**Materials and Methods:**

A retrospective, cross‐sectional study was conducted from January 2021 to October 2023, involving 2394 patients who had perioperative blood requests and underwent gynecological surgeries in the Departmental Theatres at Ramathibodi Hospital. Patient characteristics and clinical profiles, particularly the quantities of packed red blood cells (PRC) requested and utilized, were reviewed. The appropriateness of blood utilization in gynecological surgeries was calculated using standard parameters, including the cross‐match‐to‐transfusion (C/T) ratio, transfusion probability (TP), and transfusion index (TI). Descriptive, univariate, and multivariate analyses were employed.

**Results:**

Of 2394 gynecological surgeries, 431 (18%) patients had cross‐matching requests, while only 113 (4.7%) were transfused. Appropriate blood utilization was revealed by indices, with a C/T ratio of 2.43 and a TI of 0.60. 90.3% of transfusion events were administered in elective settings. Using univariable analysis, the data demonstrated that age, baseline hemoglobin, operative time, blood loss, previous abdominal surgeries, including myomectomy and endometriosis‐related surgeries, cardiovascular comorbidities, and current antiplatelet/anticoagulant use were independently associated with perioperative blood transfusion. However, only three variables significantly remained on multivariable analysis, including baseline hemoglobin (adjusted odds ratio [aOR]: 0.608; 95% confidence interval [CI]: 0.519–0.709), operation duration (aOR: 1.004; 95% CI: 1.001–1.007), and blood loss (aOR 1.003; 95% CI: 1.003–1.004).

**Conclusion:**

The study reveals appropriate blood utilization in gynecological practices. Other than in cases of active hemorrhage or anticipated extensive surgery, routine cross‐matching may be unnecessary in elective low‐risk procedures. Tailored approaches are necessary for specific indications or procedures with a high likelihood of requiring blood transfusion.

## Summary

- What's known
  ◦ Excessive perioperative blood ordering causes blood wastage.
  ◦ Previous reports showed that the Obstetrics and Gynecology Department poorly demonstrated efficiency of blood utilization.

- What's new
  ◦ Regular audits provide insight into the current situation of blood utilization during gynecological surgeries and recall strategies for improvement.

- Clinical implications
  ◦ In the tertiary care, 24‐h service is available, routine cross‐matching may be unnecessary in elective low‐risk gynecological procedures.

## 1 Introduction

Perioperative blood transfusion is a standard practice in all extensive surgical procedures, including gynecological procedures. Appropriate blood transfusion provides a safety margin for patients undergoing operations by enabling rapid reperfusion to maintain intraoperative hemodynamics in the event of an unexpected hemorrhage. Due to multiple processing steps, excessive blood requests with poor utilization will result in increased healthcare expenses, higher staff workloads, and the wastage of unused blood components [1].

Various strategic plans were implemented to address this issue. The Maximum Surgical Blood Ordering Schedule (MSBOS), a blood inventory prototype, was introduced to assist blood bank management, eliminate unnecessary preoperative tests, and reduce the number of cross‐matched blood units [2]. Studies have shown that the implementation of MSBOS reduced the C/T ratio and significantly increased monetary savings [1, 3, 4, 5], while others have given conflicting results [6, 7]. Later, the adjusted models may include institutional data, defining populations [7, 8, 9] or combining it with type and screen (T/S) approaches [10].

Perioperative blood transfusion has been reported in approximately 25.8% of elective surgeries across specialties [11]. Using standard parameters, including the cross‐match‐to‐transfusion (C/T) ratio, transfusion probability (TP), and transfusion index (TI), Zewdie et al. reported excessive blood requests with minimal transfusion in all departments performing elective surgeries [12]. The Neurosurgery Division was the most efficient in terms of blood management, while the Obstetrics Division was the least [12]. Aldaghi et al. and Misganaw et al. also reported inappropriate blood requests in several obstetric and gynecological procedures, which demonstrated by increased C/T ratio up to 9.0 [13, 14]. Inefficient blood utilization in the Obstetrics and Gynecology Department has been supported in several countries, especially in the developing world [11, 12, 15, 16]. Therefore, regular audits and the assessment of implemented policies are essential tools to evaluate the efficiency of blood utilization practices, to reduce the over‐requesting of blood reservations, and to decrease the wastage of cross‐matched blood. This study aimed to audit the appropriateness of current transfusion practices, provide recent information on transfusion rates, and provide insight into possible risk factors among patients who required blood transfusions during gynecological surgeries in an academic setting in Thailand.

## 2 Methods

### 2.1 Study Design and Patient Population

**Figure 1:**
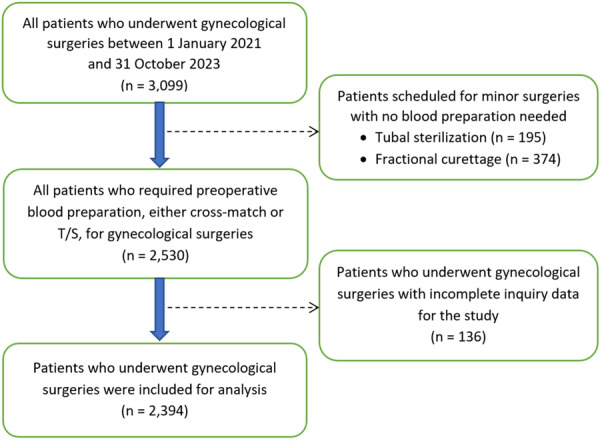
Flow diagram of patients included in the analysis after applying exclusion and inclusion criteria.

A retrospective, cross‐sectional study was conducted at the Obstetrics and Gynecology Theaters of Ramathibodi Hospital, Bangkok, Thailand. The hospital setting serves as a tertiary care referral facility for patients from other centers and the neighboring population. Gynecological consultations and surgeries, as well as the blood bank, are available throughout 24‐h services. Inclusion criteria were all patients who underwent gynecological surgeries between January 2021 and October 2023. Patients scheduled for minor surgeries with no blood preparation needed and those with incomplete medical records were excluded from the study (Figure 1). All consecutive patients who had either packed red blood cells (PRC) or T/S preparation were retrospectively reviewed.

The primary outcome was to audit the appropriateness of blood utilization practices in gynecological surgeries by using the C/T ratio, TP, and TI, standard parameters for assessment. The calculation methods and reference values were defined in Table 1 [12, 17, 18, 19, 20, 21, 22]. The secondary outcomes were to determine patient characteristics associated with blood transfusion; age, body mass index (BMI), history of previous surgeries, medical comorbidities, current antiplatelets or anti‐coagulants taking, and perioperative variables contributing to blood transfusion from previous reports, such as preoperative hemoglobin level, estimation intraoperative blood loss, operation duration, surgical procedures related malignancy, and surgical urgency. This study also examined transfusion risk assessment to distinguish between high‐risk and low‐risk patients.

**Table 1:** Parameters for blood utilization [12, 17, 18, 19, 20, 21, 22].

Parameters	Calculated formulas	Accepted value
Cross‐match‐to‐transfusion ratio (C/T)	$=\frac{\text{number}\;\text{of}\;\text{cross}‐\text{matched}\;\text{units}}{\text{number}\;\text{of}\;\text{transfused}\;\text{units}}$	< 2.5
Transfusion probability (TP)	$=\frac{\text{number}\;\text{of}\;\text{transfused}\;\text{patients}\;\times \;100}{\text{number}\;\text{of}\;\text{cross}‐\text{matched}\;\text{patients}}$	≥ 30%
Transfusion rate (TI)	$=\frac{\text{number}\;\text{of}\;\text{transfused}\;\text{units}.}{\text{number}\;\text{of}\;\text{cross}‐\text{matched}\;\text{patients}}$	≥ 0.5

Data were extracted from the hospital information system (HIS), including electronic medical records (EMR) and manual admission records. Demographic data, medical comorbidities, prior abdominal surgeries, and factors contributing to pelvic adhesions were thoroughly reviewed and documented. Preoperative risk assessment was based on variables predisposing to increased blood transfusion risk, as previously reported elsewhere [13, 23, 24, 25]. Patient characteristics and perioperative variables were evaluated for the association with PRC transfusion, including age, BMI, parity, preoperative hemoglobin level, surgical indication, type of surgical procedure (benign or malignant), operative time, intraoperative blood loss, and number of PRC units cross‐matched and transfused. Medical comorbidities included asthma, cardiovascular diseases, hypertension, diabetes, chronic kidney diseases, as well as a history of currently taking antiplatelets or anticoagulants. Previous abdominal surgeries and/or intrauterine intervention, such as cesarean section, myomectomy, and uterine curettage, were also noted for potential causes of prolonged operations and increased transfusion risk.

The preoperative transfusion risk assessment was clinically classified as low‐ or high‐risk (Table 2) based on an agreement between the anesthesiologists and gynecologists during the preoperative assessment, as per institutional policy. Low‐risk patients were anticipating blood loss during a surgical intervention requiring no more than one unit of PRC replacement (cross‐matched or T/S). Whilst the patients at high‐risk, anticipating blood loss from interventions, may require at least two units of PRC transfusion. Intraoperative blood loss was calculated by the anesthesiologist based on the weight change of the surgical swabs used and the blood content accumulating in the vacuum dispenser. Transfusion decisions by the managing team of physicians were based on clinical assessment of individual hemodynamics. Determining sample size was subjected to a 19.4% transfusion rate with 10% precision and 95% CI [23].

**Table 2:** Transfusion risk definitions.

Risk grades	Definitions
High‐risk	During preoperative assessment, identifying at least one of the following; preoperative hemoglobin less than 10 g/dL, surgical intervention related to advanced stage cancer (debulking tumor required), recurrent endometriosis (including adenomyosis and deep infiltrating endometriosis), previous abdominal or pelvic surgeries (appendectomy, myomectomy, colorectal surgeries, etc.) previous cesarean section, history of pelvic inflammatory diseases, history of cardiovascular events, current anti‐platelets or anti‐coagulants taken, and anticipating prolong surgeries to go.
Low‐risk	Other conditions do not fit in high‐risk (by default)

Approval for this study was granted by the Human Research Ethics Committee of the Faculty of Medicine Ramathibodi Hospital, in November 2023 (No. MURA2023/864). According to retrospective reviews, the IRB granted an exemption from the informed consent requirement because the study posed no risks to subjects. In addition, the waiver would not adversely affect the rights or welfare of the subjects, and the research could not practicably be carried out without the waiver. Of note, the present study was conducted in accordance with the Strengthening the Reporting of Observational Studies in Epidemiology (STROBE) guidelines for cross‐sectional studies.

### 2.2 Statistical Analysis

Baseline characteristics were summarized using frequencies and percentages for categorical variables and means with standard deviations (SD) or medians with interquartile ranges (IQR) for continuous variables, as appropriate. Comparisons between the transfusion and nontransfusion groups were performed using the chi‐square test or Fisher's exact test for categorical variables and the Student's *t*‐test or Mann–Whitney *U*‐test for continuous variables, depending on the data distribution.

Variables associated with blood transfusion at a significance level of *p* < 0.10 in univariable analyses were entered into a multivariable logistic regression model. A forward selection approach was applied to identify independent factors associated with blood transfusion, and results are presented as adjusted odds ratios (ORs) with 95% confidence intervals (CIs). All tests were two‐sided, and *p *< 0.05 were considered statistically significant. Subgroup analyses assessing the appropriateness of blood management based on transfusion risk were conducted as exploratory analyses and should be interpreted with caution. Data management and statistical analyses were performed using GraphPad Prism version 10.5.0 (GraphPad Software, Inc., La Jolla, CA, USA).

## 3 Results

During the study period, 2944 gynecological surgeries were retrieved from the hospital's HIS. Initially, 550 cases were excluded due to minor surgeries, no blood preparation needed, and data lost from the EMR. Therefore, 2394 medical records were finally eligible for the study. Of these, 18.0% (431/2394) patients requested preoperative PRC cross‐matching, but only 113 received transfusion, resulting in an overall perioperative transfusion incidence of 4.7% (113/2394). A total of 431 patients requested 629 PRC cross‐matched units, but only 259 were transfused. Using calculated parameters of blood utilization resulted in a C/T ratio of 2.43 and a TI of 0.60, as well as an audit of the appropriateness of blood preparation in gynecological surgeries (Table 3).

**Table 3:** Blood utilization in gynecological surgeries in Ramathibodi Hospital, January 2021 to October 2023.

Summary details of blood transfusion	*n* = 2394
Number of cross‐matched patients; *n* (%)	431 (18.0)
Number of transfused patients; *n* (%)	113 (4.72)
Total number of blood requests (units)	629
Total number of blood transfusions (units)	259
C/T ratio of PRC	2.43
TI of PRC	0.60
TP of PRC (%)	26.2

Abbreviations: C/T ratio, cross‐matched per transfusion ratio; PRC, packed red blood cells; TI, transfusion index; TP, transfusion probabilities.

From 2394 gynecological events recruited in this analysis, perioperative blood transfusion was significantly associated with the following preoperative risk factors: older age (47.7 vs. 43.2 years; *p* < 0.01), low baseline hemoglobin (10.8 vs. 12.6 g/dL; *p* < 0.01), no history of previous abdominal surgeries (32.7% vs. 60.5%; *p* < 0.001) especially myomectomy (9.7% vs. 3.2%; *p* < 0.01) and/or endometriosis related surgeries (8.8% vs. 3.9%; *p* = 0.02), histories of cardiovascular events (13.2% vs. 4.1%; *p* < 0.001), and antiplatelets/anticoagulants taken (8.0% vs. 2.9%; *p* < 0.01) as shown in Table 4. Intraoperative factors associated with perioperative blood transfusion included operation duration (220 vs. 145 min; *p* < 0.001), blood loss (1000 vs. 100 mL; *p* < 0.001), and oncological interventions (41.6% vs. 16.3%; < 0.001). After multivariate analysis adjusting for potential confounders, low baseline hemoglobin (aOR: 0.608, 95% CI: 0.519–0.709), prolonged operation duration (aOR: 1.004, 95% CI: 1.001–1.007), and greater intraoperative blood loss (aOR: 1.003, 95% CI: 1.003–1.004) remained independently and significantly associated with blood transfusion (Table 4). Although the transfusion rate appeared to be higher in an emergency setting, it did not reach statistical significance (9.7% vs. 5.9%; *p* = 0.10). Blood transfusion was more common in the elective setting (90.3% vs. 9.7%). Indications for PRC transfusion during gynecological surgeries are demonstrated in Table 5. Acute hemorrhage was the most predominant cause of transfusion in emergency (54.5%). Ovarian cancer (29.4%) and fibroid (26.5%) were the first and second most common indications in elective settings.

**Table 4:** Characteristics of patients who underwent gynecological surgeries in Ramathibodi Hospital, January 2021 to October 2023 (*n* = 2394).

Characteristics	Transfusion (*n* = 113)	Nontransfusion (*n* = 2281)	Odds ratio (95% CI)	*p*‐value	Adjusted odds ratio	*p*‐value
Age (years), mean (SD)	47.7 (14.4)	43.2 (12.4)	1.027 (1.012–1.042)	< 0.01	1.011 (0.993–1.030)	0.24
BMI (kg/m^2^), mean (SD)	24.6 (5.8)	24.6 (5.0)	0.999 (0.961–1.036)	> 0.99		
Baseline hemoglobin (g/dL), mean (SD)	10.8 (2.1)	12.6 (1.5)	0.598 (0.536–0.666)	< 0.001	0.608 (0.519–0.709)	< 0.001
Operation duration (min), median (IQR)	220 (176.5, 267.5)	145 (113, 190)	1.010 (1.008–1.012)	< 0.001	1.004 (1.001–1.007)	0.018
Estimated blood loss (mL), median (IQR)	1000 (700, 2000)	100 (50, 300)	1.003 (1.003–1.004)	< 0.001	1.003 (1.003–1.004)	< 0.001
Parity, *n* (%)						
Nulliparity	58 (51.3)	1250 (54.8)	1	0.50		
Multiparity	55 (48.7)	1031 (45.2)	0.869 (0.598–1.270)			
Previous surgical morbidity, *n* (%)						
No abdominal surgeries	37 (32.7)	1380 (60.5)	0.317 (0.211–0.473)	< 0.001	0.745 (0.468–1.169)	0.20
Cesarean section	14 (12.4)	452 (19.8)	0.572 (0.318–0.996)	0.05		
Myomectomy	11 (9.7)	73 (3.2)	3.262 (1.711–6.313)	< 0.01	2.232 (0.701–6.441)	0.17
Endometriosis‐related surgeries	10 (8.8)	89 (3.9)	2.391 (1.155–4.712)	0.02	2.883 (0.942–7.976)	0.06
Tuboovarian abscess	1 (0.9)	2 (0.1)	10.17 (0.696–87.80)	0.14		
Appendectomy	5 (4.4)	171 (7.5)	0.571 (0.247–1.367)	0.27		
Hysterectomy	5 (4.4)	39 (1.7)	2.661 (1.112–6.785)	0.05		
Curettage	16 (14.2)	365 (16)	0.866 (0.492–1.487)	0.69		
Other abdominal surgeries	9 (16.8)	317 (13.9)	1.252 (0.744–2.050)	0.40		
Medical comorbidities (risk of transfusion), *n* (%)						
Asthma	2 (1.8)	57 (2.5)	0.703 (0.166–2.548)	> 0.99		
Cardiovascular diseases	15 (13.2)	94 (4.1)	0.281 (0.159–0.503)	< 0.001	1.114 (0.296–3.566)	0.86
Hypertension	29 (25.7)	497 (21.8)	1.285 (0.825–1.977)	0.29		
Diabetes	15 (13.3)	214 (9.4)	1.478 (0.853–2.562)	0.19		
Chronic kidney disease	2 (1.8)	46 (2.0)	0.875 (0.206–3.234)	> 0.99		
On antiplatelets or anti‐coagulants	9 (8.0)	66 (2.9)	2.904 (1.433–5.890	< 0.01	0.999 (0.256–3.381)	0.99
Type of surgery, *n* (%)						
Gynecology	66 (58.4)	1909 (83.7)	1	< 0.001	1.776 (0.875–3.556)	0.11
Oncology	47 (41.6)	372 (16.3)	3.654 (2.467–5.391)			
Surgical urgency, *n* (%)						
Emergency	11 (9.7)	135 (5.9)	1	0.11		
Elective	102 (90.3)	2146 (94.1)	1.714 (0.926–3.171)			

**Table 5:** Indications for PRC transfusion in gynecological surgeries, Ramathibodi Hospital, Thailand, January 2021 to October 2023, (*n* = 113).

Indication of transfusion and subgroups	***n***	**%**
Group A. Emergency (*n* = 11)		
Acute hemorrhage	6	54.5
Anemia	2	18.2
Extensive surgeries and pelvic adhesions	3	27.3
Group B. Elective (*n* = 102)		
Malignancy		
Ovarian cancer	30	29.4
Uterine cancer (endometrial and sarcoma)	9	8.8
Pelvic metastasis from other cancers	5	4.9
Cervical cancer	1	1.0
Benign		
Fibroid	27	26.5
Endometriosis	14	13.7
Adenomyosis	6	5.9
Pelvic infection	4	3.9
Huge ovarian tumor	3	2.9
Anemia	1	1.0
Complications from endoscopic surgery	2	2.0

### 3.1 Blood Utilization Characteristics According to Transfusion Risk

Since elective surgeries contributed to the majority of transfusions, a subgroup analysis was conducted to assess the appropriateness of blood management based on transfusion risk determination (Table 6). The data showed a higher transfusion rate in patients from the high‐risk group than in those from the low‐risk group, as anticipated (13.2% vs. 3.3%; *p* < 0.001). These had a prevalence ratio (PR) of 4.0 (95% CI: 2.72–5.83; *p* < 0.001). Table 6 demonstrates that individual preoperative parameters, according to transfusion risk classification, can be used to differentiate high‐risk from low‐risk patients. The preoperative parameters, including baseline hemoglobin, type of surgery, previous abdominal surgeries, especially myomectomy, previous cardiovascular events, and use of antiplatelet/anticoagulants, were significantly associated with transfusion risk classification.

**Table 6:** Comparison of patient characteristics by transfusion risk (*n* = 2248).

Variables	Low risk (*n* = 1968)	High risk (*n* = 280)	*p*‐value
Age (years), mean (SD)	43.1 (12.2)	46.8 (14.7)	< 0.01
BMI (kg/m^2^), mean (SD)	24.7 (5.0)	24.0 (5.2)	0.05
Baseline hemoglobin (g/dL), mean (SD)	12.3 (1.4)	11.3 (2.1)	< 0.01
Operation duration (min), median (IQR)	147 (113,191)	163 (125, 220)	< 0.01
Estimated blood loss (mL), median (IQR)	100 (50,300)	300 (100, 600)	< 0.01
PRC transfusion, *n* (%)	65 (3.3)	37 (13.2)	< 0.001
Type of surgery, *n* (%)			
Gynecology	1653 (84.0)	195 (69.6)	< 0.001
Oncology	315 (16.0)	85 (30.4)	
Previous surgical morbidities, *n* (%)			
No abdominal surgeries	1051 (53.4)	127 (45.4)	0.25
Myomectomy	54 (2.8)	15 (5.4)	0.01
Endometriosis or ovarian cystectomy	70 (3.6)	10 (3.6)	0.72
Medical comorbidities, *n* (%)			
Cardiovascular diseases	65 (3.3)	20 (7.1)	0.001
On antiplatelets or anti‐coagulants	43 (2.2)	19 (6.8)	< 0.001

Abbreviations: BMI, body mass index; g/dL, grams per deciliter; IQR, 25% to 75% interquartile range; kg/m^2^, kilograms per square meter; min, minutes; mL, milliliters; PRC, packed red blood cells; SD, standard deviation.

Patients determined low‐risk showed significantly higher baseline Hb levels (11.4 vs. 10.0; *p* < 0.01), with a lower prevalence of gynecological malignancy interventions (16.0% vs. 30.4%; *p* < 0.001), lower rate of previous myomectomy (2.8% vs. 5.4%; *p* = 0.01), lower prevalence of previous cardiovascular events (3.3% vs. 7.1%; *p* = 0.001) and lower rate of antiplatelets/anticoagulants taken than those at high‐ risk group. Additionally, intraoperative findings confirmed that patients at high risk had longer operation durations (163 vs. 147 min; *p* < 0.001) and greater intraoperative blood loss (300 vs. 100 mL; *p* < 0.001) than those in the low‐risk group. The efficiency of PRC utilization was calculated and demonstrated in Table 7. Moreover, the probability of transfusing 1 unit of PRC per high‐risk event was higher than per low‐risk event (45.4% vs. 5.2%). Additionally, for each transfusion episode, the number of PRC units used for a high‐risk patient was approximately twice that for a low‐risk patient (3.43 vs. 1.57 units).

**Table 7:** Blood utilization indices of patients undergoing elective gynecological surgeries at Ramathibodi Hospital (*n* = 2248).

Patient risk determination	Cross‐matched patients (*n*)	Transfused patients (*n*)	Cross‐matched units (*n*)	Transfused units (*n*)	C/T ratio^a^	TP (%)^b^	TI^c^
Low risk	77	65	77	102	0.75	84.4	1.32
High risk	280	37	419	127	3.3	13.2	0.45

Abbreviations: C/T ratio, cross‐match‐to‐transfusion ratio; TI, transfusion index; TP, transfusion probability.

^a^ Number of units cross‐matched/number of units transfused; a ratio of 2.5 is suggested to indicate efficient blood usage.

^b^ Number of patients transfused/number of patients cross‐matched x100; a value of 30% indicates appropriate blood usage.

^c^ Number of units transfused/number of patients cross‐matched; a value of 0.5 indicates efficient blood usage.

Ovarian cancer and fibroids were predominantly diagnosed in patients who received blood transfusions during elective gynecological operations (Table 8). Fibroid was the most prevalent diagnosis in both high‐ and low‐risk groups. Data showed significantly different transfusion risk between patients at high‐ and low‐risk determinations if patients were diagnosed with ovarian cancer (*p* < 0.001) or fibroid (*p* < 0.01).

**Table 8:** Indications for PRC transfusion during elective settings in gynecological surgeries, Ramathibodi Hospital, Thailand, January 2021 to October 2023, (*n* = 2248).

Indications for surgeries	High risk (*n* = 243) *n* (%)	Low risk (*n* = 2005) *n* (%)	*p*‐value
Malignancy			
Ovarian cancer			
Transfusion	16 (10.3)	14 (9.0)	< 0.001
Nontransfusion	26 (16.7)	99 (64.1)	
Uterine cancer (endometrial and sarcoma)			
Transfusion	2 (1.0)	7 (10.8)	> 0.99
Nontransfusion	52 (25.14)	149 (71.0)	
Pelvic metastasis from other cancers			
Transfusion	4 (28.6)	1 (7.1)	0.30
Nontransfusion	4 (28.6)	5 (35.7)	
Cervical cancer			
Transfusion	0 (0)	1 (1.5)	> 0.99
Nontransfusion	2 (5.4)	34 (91.9)	
Benign			
Fibroid			
Transfusion	6 (0.6)	21 (2.0)	< 0.01
Nontransfusion	62 (6.0)	940 (91.4)	
Endometriosis			
Transfusion	4 (1.5)	10 (3.8)	0.08
Nontransfusion	29 (10.9)	223 (83.8)	
Adenomyosis			
Transfusion	1 (0.9)	5 (4.4)	0.28
Nontransfusion	5 (4.4)	102 (90.3)	
Pelvic infection			
Transfusion	3 (60.0)	1 (20)	> 0.99
Nontransfusion	2 (20.0)	0 (0.0)	
Huge ovarian tumor			
Transfusion	1 (1.2)	2 (2.3)	0.31
Nontransfusion	9 (10.5)	74 (86.1)	
Ovarian cyst			
Transfusion	0 (0.0)	0 (0.0)	> 0.99
Nontransfusion	8 (3.6)	215 (96.4)	
Other gynecological surgeries			
Transfusion	0 (0)	3 (2.8)	> 0.99
Nontransfusion	6 (5.6)	99 (91.7)	

*Note:* Using Fisher's Exact test to determine differences in proportions.

## 4 Discussion

The current cross‐sectional study has demonstrated the appropriateness of blood preparation practices during gynecological surgeries at an academic‐level hospital. The overall incidence of perioperative blood transfusions was 4.7%. In this series, the data have demonstrated an independent association between perioperative factors and blood transfusion in patients undergoing gynecological surgeries. After adjusting for potential confounders, the incremental transfusion rate remained significant in patients with low hemoglobin levels, longer operative durations, and greater intraoperative blood loss. Ovarian cancer and fibroids were the two most common indications observed in patients who required blood replacement in the elective setting. Acute hemorrhage, on the other hand, was the most common cause of blood transfusion identified in emergency surgeries.

The implications of these findings suggest that gynecological interventions are generally safe and rarely require blood transfusions. The transfusion rate in the current study was slightly lower than that reported by Unal et al. [11] (4.7% vs. 8.8% overall in the Gynecology/Obstetrics Department). Likewise, recent studies observed a notably low likelihood of transfusion events during gynecological surgeries, especially hysterectomy [3, 26, 27]. However, there were conflicting reports found, showing higher rates of transfusion of up to 13%–23% among gynecological surgeries [28, 29]. This discrepancy among studies may be attributed to the variations of races, socioeconomic levels, hospital standards, and institution's blood transfusion practices and policies [26]. Our institutional policy recommends correcting anemia before undergoing elective surgeries. Additionally, our study period fell within the timeframe when the number of gynecological interventions shifted from laparoscopic surgeries being an alternative to becoming the standard of care. This observation is supported by Frank et al. and Saad‐Naguib et al., who demonstrated a higher frequency of transfusion inquiry during abdominal hysterectomies (12.9%–16.5%) than its requirement in laparoscopic hysterectomies (1.7%–4.2%) [3, 27].

Over‐requesting preoperative blood reservations has frequently been cited as a cause of inappropriate management, as it increases healthcare expenses, staff workload, and the wastage of unused blood products. This was highlighted by Friedman and colleagues in the 1970s [2], and urged hospitals and countries worldwide to evaluate the efficiency of their blood management programs [12, 17, 20, 30].

Apart from the MSBOS, a blood inventory prototype introduced to improve the efficiency of blood management, the T/S approach is another model commonly recommended in programs with low transfusion rates to minimize overestimation of blood product requirements [1, 17]. Since Ramathibodi Hospital has endorsed this approach in the program for more than 20 years, this study aimed to audit the efficiency of the blood utilization practice for perioperative gynecological interventions.

Under the T/S approach, the preoperative cross‐matched PRC ordering in gynecological surgeries was reduced by more than 80%. Using a C/T ratio cut‐off of less than 2.5 and a TI value of at least 0.5, as recommended elsewhere [18], this study has demonstrated the efficiency of perioperative blood management in gynecological practices, reporting a C/T ratio of 2.43 and a TI of 0.60. However, the TP of 26.2% indicated a disproportionate number of patients who received blood transfusions compared with those who were preoperatively cross‐matched. This might indicate that we over‐recruited high‐risk patients, leading to an overestimation of blood requisitions. Two possible assumptions may explain our findings. First, it may be due to heterogeneity in the study population. As our data has shown, patients with acute hemorrhage and hemodynamic instability were the major population requiring blood transfusion in emergency settings, in contrast to a high prevalence of ovarian cancer and fibroids responsible for most of the blood transfusions in elective settings. The other reason for the overestimation of blood‐ordering may be the presence of more complex diseases in a tertiary hospital, leading the gynecologist to prefer over‐reservation rather than under‐reservation. Further studies are needed to explore this in more detail.

An ad hoc analysis was conducted comparing high‐ and low‐risk patients in elective settings, as these patients accounted for the majority of blood transfusions. The indices we used for assessment indicated efficient blood utilization in the low‐risk patients, but not in the high‐risk group. This observation verified the existing risk differences between the two groups. Apart from preoperative hemoglobin level, estimated intraoperative bleeding, and operative time, Sordia‐Hernandez et al. observed significant differences in the history of abnormal uterine bleeding and submucosal myoma between transfused and nontransfused patients who underwent hysterectomy [31]. Consistently, this study has confirmed that preoperative hemoglobin, estimated intraoperative blood loss, operative time, and the oncological proportion in gynecological procedures can be used as risk‐differentiating factors for blood utilization. As in previous reports, low preoperative mean hemoglobin levels were the most common factor contributing to blood transfusions [27, 32, 33]. The concomitant relationship between operative time and blood loss may be associated with extensive surgery. A higher percentage of oncological interventions, especially ovarian cancer, in the current study may imply surgical difficulties in elective settings, highlighting the necessity of such parameters as criteria to tailor blood transfusion risk. In addition, a high prevalence of previous abdominal surgeries, such as cesarean section, myomectomy, and endometriosis, aligns with other reports to anticipate pelvic adhesions and extensive surgeries [27, 33]. The results of our investigation have confirmed that the type of surgery, histories of previous myomectomy, cardiovascular diseases, and antiplatelet or anti‐coagulants taken, differed between the low‐risk and high‐risk groups. The results of this study further underscore that using the aforementioned parameters to identify transfusion risk is appropriate. However, we have found a low TP in high‐risk patients; this may be attributed to over‐recruitment of patients into the high‐risk group. Moreover, 90.3% of transfusion events occurred in elective settings, and a significant number of transfused patients were in the low‐risk group (65/102 in elective, Table 7), indicating that a substantial proportion of patients required transfusion at the end of the procedures. This suggests that the current institutional risk stratification criteria are highly sensitive but not specific, leading to over‐ordering of blood preparation in high‐risk patients and undetectable in some low‐risk cases. We believe a new justification model should maximize the utilization of blood resources for specific patients, such as those with ovarian cancer, fibroids, or those undergoing specific procedures.

The strength of this study lies in its provision of recent data on blood transfusion rates associated with gynecological interventions at an academic center. It confirms the need for regular audits, which support the endorsed policy and highlight areas for improvement. Apart from the anticipation of extensive surgeries and pelvic adhesion, we endorse surgical urgency as a nonnegligible factor influencing the prediction of transfusion risk. Moreover, it provides diagnostic details that prompt further research questions.

On the other hand, the study has several limitations. In a cross‐sectional audit of blood transfusion practices in gynecological surgeries, data on surgical indications, procedures, and approaches should be used with caution. Hereby, information is limited to a single tertiary hospital providing 24‐h services, including a blood bank and gynecological surgeries, and an institutional policy for preoperative blood management. We acknowledge that the study did not preclude the possibility of transfusion effects on patient outcomes, which would strengthen the findings. Finally, the different identification of an individual patient at high risk for blood transfusion in this study may vary depending on the experiences of gynecologists and/or anesthesiologists. Although no concrete criteria for risk identification were established, the population at risk in the current study may differ from that in previous reports. Further research should focus more on the homogeneity of the study population, particularly with respect to factors such as surgical urgency and specific procedures. Investigations of new strategic models are needed to overcome the low TP in high‐risk patients. In addition, identifying specific interventions for which blood requisition is necessary in the era of minimally invasive surgery is undeniable.

## 5 Conclusions

The current study audited the appropriateness of the institution's perioperative blood management practices in gynecological surgeries. Regarding the low prevalence of blood transfusion, cross‐matching may be safely avoided in low‐risk elective procedures. The information provided in this study may be limited in a tertiary center with a 24‐h blood bank and full‐service consultations and surgeries. Insufficient data to draw conclusions in emergency settings; specific procedures and indications may require tailored approaches.

## Author Contributions

**Chutima Topipat:** conceptualization, formal analysis, methodology, supervision, visualization, writing – review and editing. **Punyanuch Chulnoul:** conceptualization, data curation, investigation, methodology, resources, software, validation, writing – original draft, writing – review and editing.

## Funding

The authors received no specific funding for this work.

## Ethics Statement

Approval for this study was granted by the Human Research Ethics Committee of the Faculty of Medicine Ramathibodi Hospital, in November 2023 (No. MURA2023/864).

## Conflicts of Interest

The authors declare no conflicts of interest.

## Use of Artificial Intelligence

During manuscript preparation, the authors utilized Grammarly AI to correct grammar, enhance readability, and refine language. The final manuscript was reviewed and edited for content if needed; therefore, the authors take full responsibility for the content submitted for publication.

## Transparency Statement

The lead author Punyanuch Chulnoul affirms that this manuscript is an honest, accurate, and transparent account of the study being reported; that no important aspects of the study have been omitted; and that any discrepancies from the study as planned (and, if relevant, registered) have been explained.

## Data Availability

The datasets generated or analyzed supporting the current study are available from the corresponding author upon reasonable request. All authors have read and approved the final version of the manuscript. The corresponding author, Punyanuch Chulnoul, had full access to all of the data in this study and takes complete responsibility for the integrity of the data and the accuracy of the data analysis.
